# Understanding Infection, Viral Exacerbation and Respiratory Symptoms at Admission-Longitudinal (UNIVERSAL) study: a prospective observational cohort study protocol

**DOI:** 10.1136/bmjopen-2024-093427

**Published:** 2025-04-09

**Authors:** Tommaso Morelli, Martha Purcell, Pedro Rodrigues, Charles Roberts, Olivia Cox, Paul H Lee, Kerensa Thorne, Alexander Allen, Angelica Cazaly, Jacqueline Nuttall, James Raftery, Gareth Griffiths, Andrew Cook, Nicola White, Neil J Greening, Matthew Pavitt, James Myerson, Stefan J Marciniak, Cyrus Daneshvar, Michael G Crooks, Philip Mitchelmore, James D Chalmers, Salman Siddiqui, Karl J Staples, Tristan William Clark, Anna Freeman, Tom Wilkinson, UNIVERSAL Study group, Emma Tilt

**Affiliations:** 1 Clinical and Experimental Sciences, Faculty of Medicine, University of Southampton, Southampton, UK; 2 NIHR Southampton Biomedical Research Centre, University Hospital Southampton NHS Foundation Trust, Southampton, UK; 3 Southampton Clinical Trials Unit, University of Southampton, Southampton, UK; 4 School of Primary Care, Population Sciences and Medical Education, Faculty of Medicine, University of Southampton, Southampton, UK; 5 Department of Microbiology and Specialist Virology Centre, University Hospital Southampton NHS Foundation Trust, Southampton, UK; 6 Department of Respiratory Sciences, Institute for Lung Health, University of Leicester, Leicester, UK; 7 Department of Respiratory Medicine, University Hospitals Sussex NHS Foundation Trust, Brighton and Haywards Heath, UK; 8 Cambridge University Hospitals NHS Foundation Trust, Cambridge, UK; 9 Royal Papworth Hospital, Cambridge, UK; 10 Department of Respiratory Medicine, Plymouth Hospitals NHS Trust, Plymouth, UK; 11 Faculty of Health, University of Plymouth, Plymouth, UK; 12 Academic Respiratory Medicine, Hull York Medical School, Hull, UK; 13 Department of Respiratory Medicine, Royal Devon University Healthcare NHS Foundation Trust, Exeter, UK; 14 Division of Respiratory Medicine and Gastroenterology, Ninewells Hospital and Medical School, University of Dundee, Dundee, UK; 15 National Heart and Lung Institute, Imperial College London, London, UK

**Keywords:** SARS-CoV-2 Infection, Hospitalization, Observational Study, Patient Reported Outcome Measures, Respiratory infections

## Abstract

**Abstract:**

**Background:**

Respiratory viral infections (RVIs) are a significant cause of morbidity and hospital admission worldwide. However, the management of most viral infection-associated diseases remains primarily supportive. The recent COVID-19 pandemic has underscored the urgent need for a deeper understanding of RVIs to improve patient outcomes and develop effective treatment strategies. The Understanding Infection, Viral Exacerbation and Respiratory Symptoms at Admission-Longitudinal Study is an observational study which addresses this need by investigating the heterogeneity of RVIs in hospitalised adults, aiming to identify clinical and biological predictors of adverse outcomes. This study aims to bridge critical knowledge gaps in the clinical course and the economic impact of RVIs by characterising the phenotypic diversity of these infections and their recovery patterns following hospital admission and thus assisting with the optimal design of future interventional studies.

**Methods and analysis:**

This prospective longitudinal observational study (V.6, 20 September 2023) will be conducted across multiple UK secondary care sites from August 2022 onwards, with an aim to enrol 1000 participants testing positive for RVI. Adults admitted with respiratory symptoms who test positive for RVIs via the BioFire® FilmArray® System or other validated diagnostic PCR tests will be enrolled. The data collected include patient demographics, clinical history, comorbidities and symptoms experienced prior to, during and after hospitalisation with follow-up after discharge at weeks 1, 2, 4, 8, 12 and 26. In addition, biological samples are collected at multiple time points during the hospital stay. The primary endpoints are to study the impact of different RVIs and identify predictors of disease progression and length of stay. Secondary endpoints include time to recovery and healthcare cost. Exploratory endpoints focus on biomarker profiles associated with virus type and clinical outcomes.

**Ethics and dissemination:**

The study protocol received ethical approval from the relevant committees (English Ethics Reference Number: 22/WM/0119; Scottish Ethics Reference Number: 22-SS-0101, 20/09/2023). For patients who lack the capacity to consent, the study complies with the Mental Capacity Act 2005, using a consultee process where a family member, carer or an independent clinician may provide assent on behalf of the patient. Data from all the study centres will be analysed together and disseminated through peer-reviewed journals, conference presentations and workshops. The study group will ensure that participants and their families are informed of the study findings promptly and in an accessible format.

**Trial registration number:**

ISRCTN49183956.

**Strengths and limitations of this study**

- Multicentre design, a diverse representative UK population sample, enhances generalisability.
- Comprehensive data collection at multiple time points allows for detailed analysis of disease progression and recovery.
- Establishment of a biorepository will facilitate future research on respiratory viral infections.
- Enhance optimal design of future interventional studies.
- Reliance on UK hospital-based recruitment may limit generalisability to non-hospitalised populations and non-UK populations.

## Introduction

### Background

 Respiratory viral infections (RVIs), such as influenza, respiratory syncytial virus (RSV) and SARS-CoV-2, are frequently associated with hospital admission, particularly among vulnerable populations such as elderly individuals and those with chronic health conditions.^1^ Despite their prevalence and impact, there is a lack of detailed understanding regarding the clinical course, phenotypic variability and long-term outcomes of the wider spectrum of RVIs. Previous studies have often focused on single viral pathogens or specific patient populations, often neglecting the broader spectrum of respiratory viruses and their diverse clinical presentations.^2^ ^3 4^

The recent COVID-19 pandemic has underscored the need for research that links clinical care with rapid translation of new treatment discoveries.^5^ Understanding the natural history of acute RVIs and recovery will facilitate improved clinical management and potentially identify intervention options for those at risk of severe disease, resulting in health economic benefits.

The Understanding Infection, Viral Exacerbation and Respiratory Symptoms at Admission-Longitudinal (UNIVERSAL) observational study aims to address these critical knowledge gaps by providing a comprehensive analysis of RVIs in a diverse cohort of hospitalised adults in the UK. By capturing detailed clinical, biological and economic data across multiple time points, this study seeks to enhance our understanding of the natural history of these infections, identify biomarkers for disease severity and recovery and allow optimal design of future interventional studies.

### Objectives of the UNIVERSAL study

#### Primary objectives

- Develop phenotypic characterisation of the heterogeneous nature of acute RVI and recovery seen in patients admitted to hospital with respiratory symptoms.

#### Secondary objectives

- Enable accurate stratification of patients for the optimal design of future studies on novel pharmacological and non-pharmacological treatment strategies.
- Establish a biorepository of biological samples to facilitate endotype-level understanding of disease clusters.
- Develop an understanding of healthcare cost estimates for each patient.

#### Exploratory objectives

- Explore immune and inflammatory biomarkers associated with virus type, disease severity, recovery and length of stay to enable a precision medicine strategy.

## Methods and design

### Study design

The UNIVERSAL study is a multicentre, prospective, longitudinal, observational cohort study conducted across multiple secondary care hospitals in the UK. This multicentre approach allows the study to capture diverse patient demographics reflective of various geographic regions and healthcare settings across the UK. Participant enrolment began on 31 August 2022, with a target of 1000 RVI positive participants, and a planned end date of December 2025.

### Patient and public involvement

Patients and the public were involved in the design and planning of the UNIVERSAL study. The study team engaged with patient advocacy groups and individuals with lived experience with RVIs to ensure that the research addresses the issues most relevant to patients and their families. This involvement informed the selection of study outcomes, the development of patient information materials and the consent process and the design of the study protocol. This approach helped ensure that the data collection methods were patient-centred and minimally burdensome. This feedback helped shape the follow-up schedule of patient-reported outcome (PRO) measures.

A patient advisory group will meet with the research team during the study to review the study procedures. This group provides ongoing feedback on study materials and advises dissemination strategies to ensure that findings are communicated effectively to a lay audience. Findings from the study will be shared through accessible formats such as newsletters, public talks and social media updates, in addition to academic publications and conference presentations.

### Setting

The UNIVERSAL study is being conducted across 10 secondary care hospitals in the UK detailed in table 1. These hospitals were selected to provide a comprehensive representation of both urban and rural healthcare settings to try and enable a representative sample of the UK population. The participating hospitals are equipped with the necessary facilities and expertise to manage patients with acute RVIs, including advanced diagnostics and intensive care capabilities.

**Table 1:** UNIVERSAL recruitment centres and their recruitment timelines since August 2022

UNIVERSAL recruitment centre	Date opened to recruitment	Date closed to recruitment
University Hospital Southampton	31-Aug-2022	Recruitment ongoing
Castlehill Hospital, Hull	07-Nov-2022	May 2024
Royal Devon and Exeter University Healthcare	14-Nov-2022	June 2023
Derriford Hospital, Plymouth	10-Nov-2022	Recruitment ongoing
Addenbrooke’s Hospital, Cambridge	07-Feb-2023	February 2024
Princess Royal Hospital, Haywards Heath	07-Feb-2023	Recruitment ongoing
Glenfield Hospital, Leicester	21-Feb-2023	November 2023
Queen Elizabeth Hospital, Birmingham	23-Aug-2023	Recruitment ongoing
Royal Sussex County Hospital, Brighton	18-Sep-2023	Recruitment ongoing
St Mary’s Hospital, London	02-Oct-2023	Recruitment ongoing
Ninewells Hospital Dundee	25-Mar-2024	Recruitment ongoing

The UNIVERSAL Study is currently recruiting across several UK centres to gather comprehensive data on the spectrum of respiratory viral infections (RVIs) in hospitalised adults, covering a wide geographic area.

UNIVERSAL, Understanding Infection, Viral Exacerbation and Respiratory Symptoms at Admission-Longitudinal.

#### Participating hospitals

Table 1 provides a list of UNIVERSAL study recruitment centres and the applicable dates of recruitment. Recruitment is currently ongoing at seven active sites. Further recruitment centres are planned to open in 2024 to ensure ongoing recruitment into 2025.

#### Recruitment period

**Figure 1:**
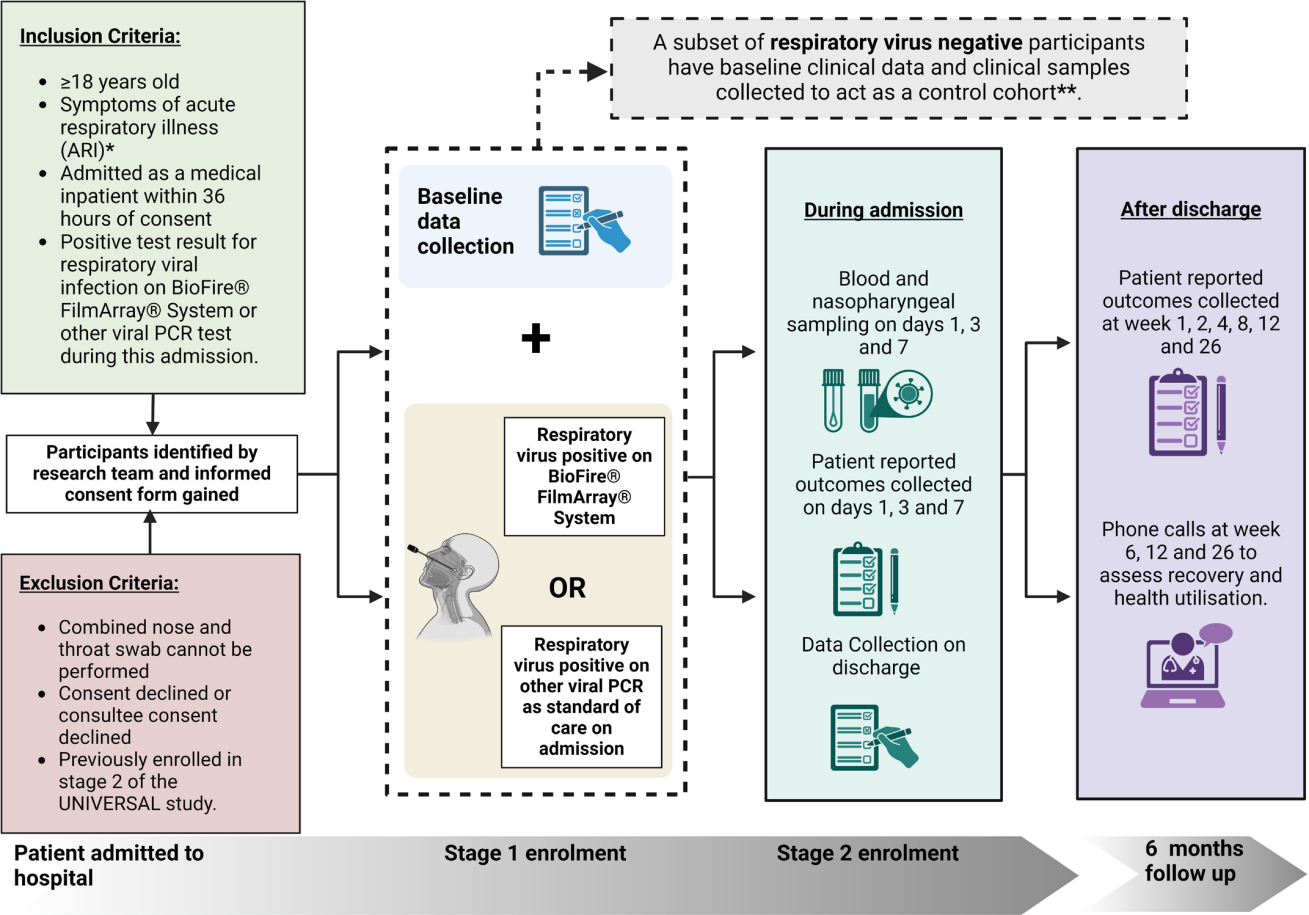
UNIVERSAL study recruitment pathway. Potential participants are identified by research staff through detailed review of the clinical records to ensure that eligibility criteria are met. Each participant is then provided with information regarding the study, and a written informed consent form is obtained. *ARI includes acute respiratory symptoms that may be caused by conditions such as acute upper or lower respiratory tract infections or acute exacerbation of chronic respiratory diseases. **Participants who test negative for RVI have baseline clinical data collected, and as per protocol amendment V.6.0 on 20 September 2023, a subset of participants testing negative for RVI have samples collected to act as a comparison cohort. Created with BioRender.com. RVI, respiratory viral infection; UNIVERSAL, Understanding Infection, Viral Exacerbation and Respiratory Symptoms at Admission-Longitudinal.

Recruitment for the UNIVERSAL study began in August 2022 and will continue until 1000 RVI positive participants are recruited. The recruitment process involved identifying eligible patients who meet the inclusion criteria on admission to the participating hospitals with respiratory symptoms, figure 1. This study aims to enrol a total of 1000 RVI-positive patients, capturing a broad spectrum of RVIs. Participants who test negative for RVI have baseline clinical data collected, and as per protocol amendment V.6.0 on 20 September 2023, a subset of participants testing negative for RVI have samples collected to act as a control group.

### Participants

#### Eligibility criteria

Eligible participants for the UNIVERSAL study are identified on their admission to the hospital with respiratory symptoms. To qualify for the study, individuals must meet specific inclusion criteria at the time of screening. The eligibility criteria, recruitment process and participant pathway through the study are detailed in figure 1.

To be eligible, participants must be aged 18 years or older and exhibit features of an acute respiratory illness. This includes conditions such as acute upper or lower respiratory illnesses (eg, rhinitis, rhinosinusitis, pharyngitis, pneumonia, bronchitis and influenza-like illness) or an acute exacerbation of a chronic respiratory illness (such as chronic obstructive pulmonary disease (COPD), asthma or bronchiectasis). The diagnosis must be confirmed by a treating clinician. Additionally, participants must have been admitted as medical inpatients within the past 36 hours, with the admission time defined as the point at which the decision was made to admit the patient rather than the time of their arrival at the hospital.

Furthermore, participants must have a positive test result for RVI using the BioFire® FilmArray® System or other NHS Trust approved diagnostic PCR test during the current admission to be enrolled in the virus positive arm. A range of viral PCR platforms are employed by NHS Trusts in the UK which detect common respiratory viral pathogens including RSV, influenza and SARS-CoV-2. If on admission, a potential participant has tested negative using a limited respiratory virus PCR panel, once consented to UNIVERSAL, a respiratory tract swab sample is obtained and run on the BioFire® FilmArray® System to detect an extended panel of respiratory pathogens, including influenza A and B, RSV, SARS-CoV-2, parainfluenza viruses 1–4, human metapneumovirus, rhinovirus/enterovirus, adenovirus and coronaviruses 229E, NL63, OC43, HKU1. This ensures that a broad range of respiratory viral pathogens are included in the study. Participants who test negative for RVI have baseline data collected only. Participants are excluded from the study if they meet any of the following criteria: they or their consultee decline consent, they cannot undergo combined nasal and throat swabbing due to either personal refusal or a medical contraindication. Participants enrolled in the UNIVERSAL study who test positive for RVI, and complete study procedures cannot be enrolled a second time.

#### Virus-negative comparison cohort

While the primary focus of the UNIVERSAL study is on hospitalised patients who test positive for a respiratory virus using the BioFire® FilmArray® System, a subset of virus-negative patients (n=100) is also being recruited (as per protocol amendment V.6.0, 20 September 2023). This group consists of individuals who present with respiratory symptoms but test negative for all viruses. This comparison cohort will allow for exploratory analyses of differences in clinical characteristics and outcomes between patients with confirmed viral infections and those in whom no virus is detected. All virus-negative participants provide written informed consent (see online supplemental material 1).

#### Selection of participants

The selection process involves a thorough review of medical records and clinical assessments to confirm eligibility based on the inclusion and exclusion criteria (figure 1). Trained research staff approach potential participants, providing detailed information about the study, including its objectives, procedures, risks and potential benefits. This ensures that participants can make an informed decision about their involvement in the study.

#### Consent procedure

All consent procedures adhere to ethical guidelines and relevant regulations, ensuring that participants’ rights and welfare are protected throughout the study. Participants with the capacity to consent are provided with detailed information about the study, including its purpose, procedures, risks and potential benefits. Written informed consent by completion of the informed consent form (online supplemental material 1) is obtained before any study-specific procedures are conducted.

The study complies with the Mental Capacity Act 2005. If a participant lacks the capacity to consent, a personal consultee such as a family member, carer, or friend may be approached to provide assent.

In cases where a personal consultee is not available, a nominated consultee may be sought. A nominated consultee is an independent person, usually a healthcare professional not connected to the research team, who can provide advice on whether the participant should join the study.

The personal/nominated consultee will be advised to set aside their own views and take into consideration the patient’s wishes and interests. Advance decisions and statements made by the patient about their preferences and wishes will always take precedence.

In the event of the patient recovering capacity following enrolment by the consultee, the patient will be asked to read the participant information sheet and provide consent for themselves. The patient may give consent, withdraw but have data and/or samples collected so far retained or withdraw and have their data and/or samples destroyed.

#### Participant withdrawal

The participant, or their consultee where a participant lacks capacity, is free to withdraw consent from the study at any time without providing a reason, and with no detriment to their medical care or legal rights. Investigators may withdraw a patient from the study in the interests of participant safety or the integrity of the research study.

### Data collection

**Figure 2:**
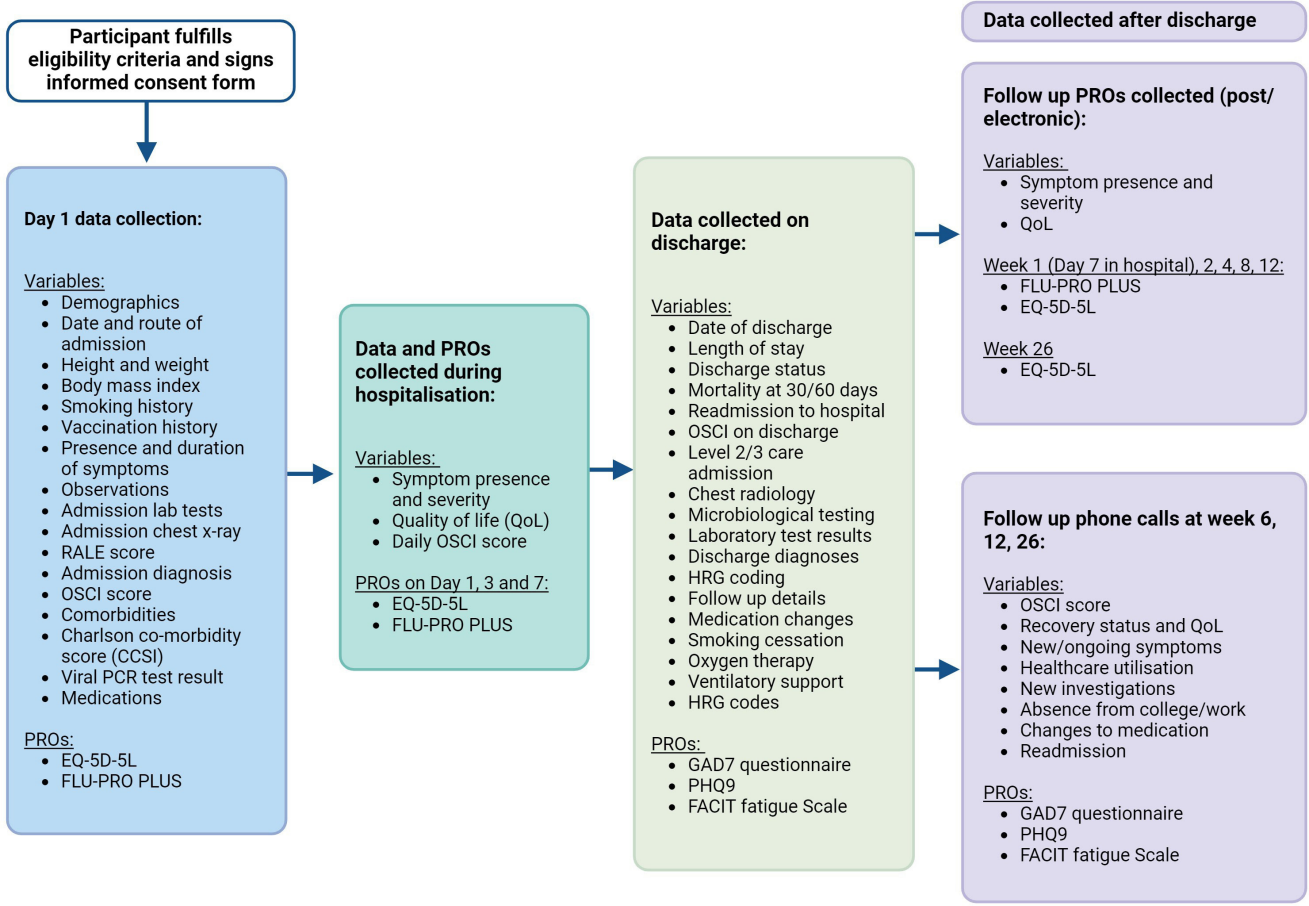
Data capture and timepoints for participants in the UNIVERSAL study. EQ-5D-5L, EuroQol 5-Dimension 5-Level; FACIT, Functional Assessment of Chronic Illness Therapy—Fatigue Scale; FLU-PRO PLUS, inFLUenza Patient-Reported Outcome Plus; GAD-7, Generalised Anxiety Disorder-7; HRG, Healthcare Resource Group; OSCI, Ordinal Scale for Clinical Improvement; PHQ-9, Patient Health Questionnaire-9; PRO, patient-reported outcome; RALE, Radiographic Assessment of Lung Edema; UNIVERSAL, Understanding Infection, Viral Exacerbation and Respiratory Symptoms at Admission-Longitudinal. Created with BioRender.com.

Data will be collected at multiple time points: at admission, during hospitalisation and post-discharge. This comprehensive approach allows for detailed tracking of disease progression and recovery. The following sections outline the clinical data collected at each stage of the study (figure 2 and online supplemental material 2).

#### Baseline data collection (on admission)

On admission, detailed demographic information, medical history, clinical presentation and initial laboratory and imaging results are recorded. The initial treatment regimen is also documented.

#### In-hospital data collection

During hospitalisation, daily clinical assessments, including Ordinal Scale for Clinical Improvement (OSCI) score, oxygen requirements, symptom progression, repeated laboratory tests, follow-up imaging studies, treatment updates, complications and outcomes such as length of stay and discharge status, will be recorded.

#### Postdischarge data collection

Follow-up assessments are conducted at 1 week, 2 weeks, 4 weeks, 8 weeks, 12 weeks and 26 weeks postdischarge. These assessments monitor long-term recovery, including symptom resolution, new or persistent symptoms, quality of life, readmissions, long-term outcomes and ongoing medical needs and treatments. Data are gathered to analyse healthcare resource utilisation and time off from normal activities, specifically work and education.

#### Methods of follow-up

Follow-up methods include telephone calls and electronic surveys or postal methods, depending on the participant’s preference and logistical considerations. This thorough and structured follow-up schedule ensures detailed monitoring of participants’ recovery and long-term health outcomes, providing valuable insights into the natural history and economic impact of RVIs.

### Sample collection and Biobanking

All UNIVERSAL participants consent to provide biological samples as part of the study procedures. Samples are taken on day 1, 3 and 7 of enrolment while participants remain in hospital as detailed in online supplemental material 2. The biological samples provided include blood, upper respiratory tract swabs and nasal lining fluid. After initial storage at individual recruitment centres, samples are transferred to a secure UK biobanking facility for longer-term storage. Biological samples are anonymised and can be linked to clinical data collected as part of the study using anonymised participant identification numbers. In line with study objectives, this provides a biorepository of clinical samples to allow the exploration of inflammatory biomarkers associated with virus type and disease severity.

### Variables

#### Primary outcomes

The primary outcomes of the UNIVERSAL study are focused on characterising the clinical impact of RVIs in hospitalised adults. This includes not only documenting the occurrence of various respiratory viruses, including influenza, RSV and SARS-CoV-2, but also a broader range of viruses such as rhinoviruses, parainfluenza viruses and human metapneumovirus. The study aims to evaluate their association with disease severity, progression and recovery. The study also aims to identify clinical and biological predictors of these outcomes, including markers such as viral load, inflammatory markers such as C reactive protein (CRP) and patient demographics. Another key outcome is measuring the duration of hospital stay for patients with RVIs and understanding the factors that contribute to prolonged hospitalisation.

#### Secondary outcomes

Secondary outcomes provide a broader understanding of the long-term effects and economic impact of RVIs. These include evaluating the time required for patients to recover from their acute respiratory illness, defined by the resolution of symptoms and normalisation of clinical markers. The study also aims to analyse the direct and indirect costs associated with RVIs, covering hospitalisation costs, outpatient visits, medication expenses and loss of time from normal activities. Additionally, the study seeks to identify biomarker profiles associated with different clinical outcomes, which will aid in the development of precision medicine approaches.

#### Exposure—RVI

The primary exposure in the study is the presence of a confirmed RVI in hospitalised adults with acute upper or lower respiratory illnesses (eg, rhinitis, rhinosinusitis, pharyngitis, pneumonia, bronchitis and influenza-like illness), including acute exacerbations of chronic respiratory illnesses (such as COPD, asthma or bronchiectasis). This is determined using the BioFire® FilmArray® System or other validated clinical diagnostic PCR-based methods established at clinical sites during the patient’s current hospital admission.

The diagnostic criteria for RVIs in the UNIVERSAL study are based on laboratory confirmation using the BioFire® FilmArray® System or other PCR tests. Clinical diagnoses made by treating clinicians, supported by symptoms and imaging findings, are also used to categorise respiratory illnesses.

#### Predictors

The study examines a range of predictors that may influence the primary and secondary outcomes. These predictors include demographic factors such as age, sex, ethnicity and socioeconomic status. Clinical history, including pre-existing comorbidities such as COPD, asthma, cardiovascular disease, smoking status and vaccination history, is another important predictor. Clinical presentation at the time of admission, including symptoms, severity scores such as the National Early Warning Score 2 (NEWS2)^6^ and initial laboratory and imaging findings, are also critical predictors.

By clearly defining these variables, the UNIVERSAL study aims to provide comprehensive insights into the phenotypic diversity, disease progression and economic impact of RVIs in hospitalised adults. This detailed analysis will contribute to the development of targeted interventions and improve patient outcomes

### Data sources and measurement

The UNIVERSAL study employs rigorous and standardised data collection methods to ensure consistency and reliability across multiple sites. For each variable of interest, the sources of data and methods of assessment are carefully outlined below and in online supplemental material 2. Where there are multiple groups or measurement methods, comparability is ensured through standardised protocols and training.

#### Demographic and clinical data

Demographic and clinical data are sourced from patient interviews and medical records. Detailed demographic information, including, sex, age at enrolment, height, weight, ethnicity, postcode, number of people in household and smoking history, is collected through structured patient interviews conducted by trained research staff. Medical history, including pre-existing comorbidities and vaccination history, is obtained from patient interviews and verified against medical records. Clinical presentation data, encompassing symptoms, duration of illness and severity scores such as the NEWS2 and OSCI, are documented using standardised assessment tools. Length of stay is addressed through review of clinical discharge summaries. During the discharge visit, details of treatments received during admission including time spent on intensive care or high dependency are collected from medical records. Mortality and readmission data are collected through medical records and patient interviews. While socioeconomic factors are not a primary focus of the study, participant postcodes are collected to allow for area-based deprivation analyses (eg, linkage to the Index of Multiple Deprivation).

#### Laboratory and imaging data

RVIs are confirmed using the BioFire® FilmArray® System or other PCR-based methods, with results recorded in the electronic study database. Levels of inflammatory markers, such as CRP are measured through blood tests conducted at baseline and repeated at discharge. Chest X-ray imaging data in the form of the reports issued by qualified radiologists are captured using standardised interpretation criteria.

#### Patient-reported outcomes

PROs are collected using validated questionnaires administered during follow-up visits. Symptom resolution is assessed by asking patients to report the presence and severity of symptoms at each follow-up visit through completion of the FLU-PRO-Plus.^7^ Quality of life is measured using standardised tools, such as the FLU-PRO-Plus and EQ-5D-5L,^8^ to capture the patient’s perspective on their recovery. Once discharged from hospital, recovery is assessed using standardised questionnaires, listed in table 2, for symptom severity, quality of life, fatigue, depression and anxiety. This provides a comprehensive and holistic approach to monitoring patient recovery, the importance of which has been highlighted following the SARS-CoV-2 pandemic. Details of the PROs used as part of the UNIVERSAL study are provided in table 2.

**Table 2:** Patient-reported outcomes (PROs) in the UNIVERSAL study

PRO	Variable	Format	Reference
FLU-PRO-Plus	Symptom severity and resolution in viral illness	Assesses severity of symptoms in viral illness using 32 items across six body systems scored on a five-point severity scale based on recall of the previous 24 hours. Additional questions include loss of taste and smell.Provides a total score and individual body system scores of symptom severity and recovery over time.	^7^
EQ-5D-5L	Functional health status and quality of life (QoL)	Health status is assessed across five dimensions—mobility, self-care, usual activities, pain and anxiety/depression with five levels of severity in each dimension. A visual analogue scale rates health from 0 (worst health imaginable) to 100 (best health imaginable).	^8^
PHQ-9	Depression	Assesses for depressive symptoms and tracks changes over time. Consists of nine questions, corresponding to the 9 DSM-IV criteria for depressive disorders. Responses to each question are scored using a four-point severity scale 0 (not at all) to 3 (nearly every day). The combined total score indicates the severity of depression.	^14^
GAD-7	Anxiety	Measures the severity of generalised anxiety. Consists of seven questions that assess frequency of anxiety-related symptoms over the previous 2 weeks. Responses to each question are scored using a four-point severity scale 0 (not at all) to 3 (nearly every day). The combined total score indicated the severity of anxiety.	^15^
FACIT-Fatigue scale	Fatigue	A component of the Functional Assessment of Chronic Illness Therapy (FACIT) measurement system designed to assess the level of fatigue experienced by individuals, particularly those with chronic illnesses. It evaluates the impact of fatigue on QoL. The scale consists of 13 items addressing aspects of fatigue over the previous 1 week. Each item is rated on a 5-point Likert scale. The scores are summed with higher scores indicating less fatigue and better functional status.	^16^

The UNIVERSAL Study collects a variety of patient-reported outcomes (PROs) at multiple timepoints, from admission to 6 months post-discharge, to objectively assess symptoms and health-related quality of life (QoL). This table provides an overview of the PROs used in the study, including the specific variables they assess, their formats and the relevant references for each outcome measure.

EQ-5D-5L, EuroQol 5-Dimension 5-Level; FACIT, Functional Assessment of Chronic Illness Therapy—Fatigue Scale; FLU-PRO-Plus, inFLUenza Patient-Reported Outcome Plus; GAD-7, Generalised Anxiety Disorder-7; PHQ-9, Patient Health Questionnaire-9; UNIVERSAL, Understanding Infection, Viral Exacerbation and Respiratory Symptoms at Admission-Longitudinal.

#### Economic data

Economic data are sourced from healthcare utilisation records and patient interviews. Direct costs are obtained from HRG codes, while indirect costs, such as time off work or education, are estimated through patient interviews and self-reported data.

#### Data management

Participant data are collected at individual recruitment centres and retained in accordance with current data protection regulations. Participant data are pseudo-anonymised by assigning participant identifier codes used to identify the participant during the study and to facilitate participant-related clarification between sites and Southampton Clinical Trials Unit (SCTU). Each individual site retains a participant identification code list which is only available to site staff.

Data are entered onto a secure centralised electronic database as per an electronic case report form only by trained personnel with specific authorisation to access study data. Research data are stored in an anonymised format under the participant identifier number so that participants are not identifiable, and confidentiality is maintained. Any data collected as part of the trial will be securely stored in line with the Data Protection Act and General Data Protection Regulations.

The study database is monitored and audited continuously by a data management team at the SCTU. A data management plan (DMP) outlines the study-specific data management strategy. Central monitoring of study activities and milestones is overseen by the SCTU. Data are checked for missing or inconsistent values, and any discrepancies are raised as queries with individual sites electronically. Sites then respond to queries to resolve the discrepancies, and appropriate amendments are made on the study database. In this way, missing or incomplete data items are identified promptly to ensure data quality and completeness. The SCTU facilitates the collection of the PROs at study timepoints (weeks 1, 2, 4, 8, 12 and 26) either electronically or by postage of paper forms as per participant preference. Follow-up visits at weeks 6, 12 and 26 are carried out via telephone by trained research staff at individual recruitment centres, and data are entered onto the study database.

Once the participant has completed the study procedures after 6 months, the end of study form is completed. At the end of the study, when all participants have completed 6 months follow-up and all data queries have been resolved, the database will be frozen, and data archived according to SCTU policy.

### Bias

To mitigate potential sources of bias in the UNIVERSAL study, several strategies are employed. Standardised protocols for data collection and measurement are used across all participating sites, ensuring consistency and reliability of the collected data. All research staff undergo training to adhere to these protocols, which helps minimise variability in the data collection procedures. Additionally, quality control checks and audits are conducted to identify and correct any discrepancies or errors. This includes reviewing data for completeness and accuracy, as well as cross-verifying information from different sources.

A centralised electronic database with secure access is used to maintain data integrity and confidentiality. Efforts are also made to minimise selection bias by ensuring a broad and representative sample of the patient population from diverse urban and rural settings. Moreover, the study employs rigorous inclusion and exclusion criteria to ensure that the patient population is well-defined and comparable across sites.

### Study size

The study size for the UNIVERSAL study was determined based on several considerations. Based on previous studies, a sample size of 1000 patients is believed to provide sufficient power to detect significant differences in primary outcomes. This calculation was based on expected variability in outcomes based on previous studies.^4 9 10^ The sample size aims to ensure that the findings are statistically robust and clinically meaningful. By recruiting a large cohort across multiple geographical UK hospital sites, the study seeks to achieve comprehensive and generalisable results that can inform clinical practice and policy decisions. The sample size calculation also considered potential dropouts and loss to follow-up, ensuring that the final dataset remains sufficiently powered for detailed analyses.

### Sample size calculation

Based on previous studies and expected variability in outcomes, a sample size of 1000 patients is estimated to provide sufficient power to detect significant differences in primary outcomes for the most prevalent respiratory viruses.^9 10^ In terms of descriptive statistics on the incidence/prevalence rates, 1000/2000 participants would conservatively allow the estimation with a 95% CI of ±3.1%/2.2%. There is little consensus in the literature on how best to calculate a formal sample size for cluster analysis such as latent class modelling. Simulation studies have suggested that sample sizes in the order of 500–1000 participants are usually sufficient even in the presence of weak class-indicator associations for 80% power or greater.^9 10^ To develop a clinical prediction model of progression of illness, a minimum sample size of 932 would be required. This assumes that for a model to be useful, it would need to have an area under the receiver operating characteristic (ROC) curve of at least 0.85 and is based on an outcome prevalence of 10% and up to 18 parameters in the final model.

### Statistical methods

A detailed statistical analysis plan has been designed for the UNIVERSAL study involving all statistical considerations to ensure robust and reliable results. Here, we will only describe the essential statistical methods for the primary analysis. For analysis involving multiple comparisons, we will adjust them with Bonferroni correction. SAS V.9.4 or above, STATA V.18 or above and R V.4.4 or above will be used for analyses.

Descriptive statistics will be used to summarise baseline characteristics, clinical presentation and outcomes related to different viral pathogens. Measures of central tendency (mean, median) and dispersion (SD, IQR) will be calculated for continuous variables, while frequencies and percentages are used for categorical variables. Univariate analyses will be used to explore the relationship between key characteristics and the outcome measures. Clustering techniques will be used to explore similar patterns with respect to symptoms, severity and duration.

Multivariable regression models will be employed to identify associations between key predictors and outcomes while controlling for potential confounders. These models will be adjusted for demographic factors (age, sex, ethnicity, socioeconomic status, vaccination status), clinical variables (severity scores, comorbidities) and treatment regimens. Prior to model fitting, multicollinearity among covariates will be assessed using appropriate statistical methods (eg, variance inflation factor), and adjustments will be made if necessary to ensure model stability. The choice of regression model (eg, logistic regression for binary outcomes, linear regression for continuous outcomes) depends on the nature of the dependent variable. For analysis involving data at multiple time points, multilevel regression with first-level autoregressive error correlation structure (ie, AR(1)) will be used to control for within-subject correlation.

#### Subgroup and interaction analyses

Subgroup analyses are conducted to explore differences in primary and secondary outcomes among specific patient groups, such as those with different respiratory viruses (eg, influenza, RSV, SARS-CoV-2) or varying severity levels. Interaction terms are included in regression models to examine potential interactions between key variables, such as the interaction between age and comorbidities in predicting disease severity. We will also consider stratifying the analysis by different respiratory viruses if the sample size allows. These analyses help identify variations in treatment effects and outcomes across different subpopulations.

#### Missing data

Missing data are handled using multiple imputation techniques to ensure that the analyses remain robust and unbiased. This approach involves creating several imputed datasets where missing values are estimated based on observed data. Specifically, we will use the Additive Regression, Bootstrapping and Predictive Mean Matching (*aregImpute*) function in *R* package *Hmisc*^11^ to conduct at least five multiple imputations. The results from these datasets are then combined to produce final estimates. Sensitivity analyses are performed to assess the impact of analysis methods with and without imputation on the primary study findings.

#### Loss to follow-up

Loss to follow-up is addressed through diligent follow-up procedures, including regular contact with participants via phone calls, electronic surveys and in-person visits, as laid out in the SCTU DMP. The reasons for and extent of loss to follow-up are documented, and statistical methods, such as missing data imputation described above, are used to account for the impact of censored data on study outcomes. This helps maintain the integrity of the dataset and ensures valid and reliable results.

### Ethics and dissemination

#### Ethical and safety considerations

The UNIVERSAL study has received ethical approval from relevant ethics committees, including the English Ethics reference number 22/WM/0119 and the Scottish Ethics reference number 22-SS-0101. Informed consent is obtained from all participants or their consultee before enrolment. For participants lacking capacity, consent is sought from a personal or nominated consultee. The study adheres to Good Clinical Practice (GCP) guidelines and current data protection regulations, ensuring the ethical conduct of research and the protection of participant rights.

#### Monitoring and study oversight

The study is coordinated by the SCTU and oversight is maintained by a Study Management Group chaired by the Chief Investigator. The study may be subject to inspection and audit by University Hospital Southampton NHS Foundation Trust (under their remit as Sponsor), SCTU (as the Sponsor’s delegate) and other regulatory bodies to ensure adherence to the principles of GCP, Research Governance Framework for Health and Social Care, applicable contracts and national regulations.

#### Dissemination plan

The findings from the UNIVERSAL study will be disseminated through multiple channels to ensure wide accessibility and impact. This includes publications in peer-reviewed scientific journals and presentations at national and international conferences. If the participant agrees to receiving the information, patients or carers will be notified of the results of the study, in an appropriate format and language suitable for lay members. The study will be registered on a publicly available database that will be regularly updated throughout the life of the study and will include the final report when available.

## Discussion

This novel study builds on work in asthma^12^ and COVID-19^13^ and represents the first large-scale, detailed observational study of severe RVI in the UK in the post-COVID-19 era. The diverse demographic and clinical data collected in this study offer valuable insights into the epidemiology of RVIs in the UK, with the wealth of granular data expected to highlight the significant variability in clinical presentations and outcomes. The inclusion of various respiratory viruses allows for comparative analyses that can inform both clinical practice and public health strategies.

One of the key strengths of the UNIVERSAL study is its comprehensive approach to data collection, encompassing detailed laboratory and imaging studies, as well as PROs. This multifaceted dataset enables the identification of clinical and biological predictors of disease progression, providing a basis for developing targeted interventions and improving patient management.

Efforts to minimise bias through standardised protocols and rigorous training of research staff ensure the reliability and validity of the findings. Additionally, the use of advanced statistical methods to control for confounding factors and handle missing data enhances the robustness of the analyses.

However, the study is not without limitations. The reliance on hospital-based recruitment may limit the generalisability of the findings to non-hospitalised populations. Furthermore, despite efforts to minimise loss to follow-up, some degree of attrition is inevitable, which could impact the completeness of long-term outcome data.

The UNIVERSAL study is poised to make significant contributions to the understanding of RVIs in hospitalised adults. The detailed data collected across multiple sites provide a comprehensive picture of the clinical and economic burden of these infections. The findings from this study will inform the development of precision medicine approaches, tailored interventions and more effective management strategies, ultimately improving outcomes for patients with RVIs.

## Supplementary material

10.1136/bmjopen-2024-093427 online supplemental file 1

10.1136/bmjopen-2024-093427 online supplemental file 2
