# Supplementary material for: Understanding Infection, Viral Exacerbation and Respiratory Symptoms at Admission-Longitudinal (UNIVERSAL) study: a prospective observational cohort study protocol
Source: BMJ Open. 2025 Apr 9;15(4):e093427. doi: 10.1136/bmjopen-2024-093427 (PMC11987089; doi:10.1136/bmjopen-2024-093427)
Supplement: online supplemental file 1 [file bmjopen-15-4-s001.pdf]

TO BE PRINTED ON LOCAL HEADED PAPER

UNIVERSAL Study: Understanding Viral infection and Respiratory Symptoms following admission to hospital

Registration ID:

Patient ID*: **Name:**

***only for those who go onto provide samples**

To be completed by a participant who is able to consent at the time of study entry.

Please *initial* each box

1. I confirm that I have read (or had read to me) the Patient Information Sheet [version ………, dated ……………………….…] for the above study and I fully understand what is involved in taking part in this trial. I have had the opportunity to ask questions, and these have been answered satisfactorily.

2. I understand that my participation is voluntary and that I am free to withdraw at any time, without giving a reason and without my medical care or legal rights being affected.

3. I understand that relevant sections of my medical records, and data collected during the study, may be looked at by individuals from the Sponsor or their delegates, from regulatory authorities, or from the NHS Trust where it is relevant to my taking part in this research. I give permission for these individuals to have access to my records.

4. Access to my medical information: I agree that medical information collected by the doctors and hospitals which provide me with care and which may be located in local or national health and research organizations (including hospital admission, civil registration, audit and research data) may be provided to the study coordinating centre both during and for up to 10 years after the scheduled follow-up period. I understand that information that identifies me will be passed securely to such bodies to make this possible and that I can opt out of this at any time by writing to the coordinating centre team.

5. I understand that information about me (including name, email, postcode and phone number) and my progress in the study will be recorded on a computer database, and that this data will be stored on computers supervised by the University of Southampton. I understand that this information will be kept securely and confidentially.

6. I give permission for a copy of this consent form to be sent to the Southampton Clinical Trials Unit (where it will be kept securely), to allow confirmation of my consent.

7. I agree that the blood samples, swab samples and information collected about me will be stored on behalf of the UNIVERSAL Trial Management Group and may be used in future ethically approved projects. I understand that some of these projects may be carried out by researchers other than the UNIVERSAL Trial Management Group and that the samples may be sent for testing

(including genetic testing) outside of the UK (for example the European Economic Area or the USA).

8. I consent to my pseudonymised trial data being used in future research by Janssen Pharmaceuticals, Synairgen plc or by third parties (e.g. Leidos Biomedical Research) involved in this research, including those both inside and outside the UK (for example Europe or the USA).

9. I agree to my General Practitioner being informed of my participation in the study.

10. I understand that the sponsor of this trial is the University Hospital Southampton NHS Foundation Trust.

11. I agree to take part in the **UNIVERSAL** study

OPTIONAL:
The following part of the UNIVERSAL study is optional. Please initial to indicate whether you agree.

1. I agree to provide additional blood samples for the biomarker analysis component of the study as described in the patient information sheet for the above study

2. I agree to give an extra nose/throat swab sample for storage to be used in future research as described in the patient information sheet for the above study

3. I agree to take part in the **UNIVERSAL** Sub-study titled “Virus Negative Sub-Study”

4. **The EQUATE study** - I would consider discussing my views on the use of digital technology in research. I agree for my contact details and demographic information (e.g., age, gender, ethnicity) to be shared so I can be contacted by a researcher at the University of Southampton.

………………………………	………………………………	……../……../…………
PRINTED name of participant	Signature	Today’s date
…………………………………	………………………………	……../……../…………
PRINTED name of person receiving consent	Signature	Today’s date

REMINDER FOR RESEARCH TEAM: **Original or copy signed consent form in Investigator Site File; copy given to the patient; copy filed in the patient's medical records; One copy emailed to SCTU via secure nhs.net address, encrypted email or SafeSend**
