# Supplementary material for: Understanding Infection, Viral Exacerbation and Respiratory Symptoms at Admission-Longitudinal (UNIVERSAL) study: a prospective observational cohort study protocol
Source: BMJ Open. 2025 Apr 9;15(4):e093427. doi: 10.1136/bmjopen-2024-093427 (PMC11987089; doi:10.1136/bmjopen-2024-093427)
Supplement: online supplemental file 2 [file bmjopen-15-4-s002.docx]

| **Supplementary Material 2: Study procedures, timepoints and data sources for the UNIVERSAL Study** | | | | | | | | | | | | | | |
| --- | --- | --- | --- | --- | --- | --- | --- | --- | --- | --- | --- | --- | --- | --- |
| **Study visit** | **Visit 1** | **Visit 2** | **Visit 3** | **Visit 4** |  |  | **Visit 5** |  | **Visit 6** | **Visit 7** |  | **Data source** | | |
| **Time (days):** | **Day 1** | **Day 3** | **Day 7** | **Discharge** | **Week 2** | **Week 4** | **Week 6** | **Week 8** | **Week 12** | **Week 26** | **Readmission** | **Participant** | **Medical records** | **BioFire® FilmArray® System** |
| **Window** |  | **+/- 1 day** | **+/- 1 day** | **+/- 3 days** |  |  |  |  |  |  |  |  |  |  |
| Demographics |  |  |  |  |  |  |  |  |  |  |  |  |  |  |
| Sex at birth (male/female) | **X** |  |  |  |  |  |  |  |  |  |  | **X** | **X** |  |
| Date of birth and age | **X** |  |  |  |  |  |  |  |  |  |  | **X** | **X** |  |
| Admission height (m) and weight (Kg) | **X** |  |  |  |  |  |  |  |  |  |  | **X** | **X** |  |
| BMI (calculated) | **X** |  |  |  |  |  |  |  |  |  |  |  | **X** |  |
| Number of people in household | **X** |  |  |  |  |  |  |  |  |  |  | **X** | **X** |  |
| Smoking history (current/past/never) | **X** |  |  |  |  |  |  |  |  |  |  | **X** | **X** |  |
| Vaping history | **X** |  |  |  |  |  |  |  |  |  |  | **X** | **X** |  |
| Ethnicity | **X** |  |  |  |  |  |  |  |  |  |  | **X** | **X** |  |
| Pregnant | **X** |  |  |  |  |  |  |  |  |  |  | **X** | **X** |  |
| Healthcare worker | **X** |  |  |  |  |  |  |  |  |  |  | **X** | **X** |  |
| Care/nursing home resident | **X** |  |  |  |  |  |  |  |  |  |  | **X** | **X** |  |
| Vaccination history |  |  |  |  |  |  |  |  |  |  |  |  |  |  |
| Influenza vaccination (current season) | **X** |  |  |  |  |  |  |  |  |  |  | **X** | **X** |  |
| SARS-CoV-2 vaccination (number) | **X** |  |  |  |  |  |  |  |  |  |  | **X** | **X** |  |
| Pneumococcal vaccination | **X** |  |  |  |  |  |  |  |  |  |  | **X** | **X** |  |
| Admission data and presenting History |  |  |  |  |  |  |  |  |  |  |  |  |  |  |
| Date of admission | **X** |  |  |  |  |  |  |  |  |  | **X** |  | **X** |  |
| Presence and duration of symptoms | **X** |  |  |  |  |  |  |  |  |  | **X** | **X** |  |  |
| Primary diagnosis | **X** |  |  | **X** |  |  |  |  |  |  | **X** |  | **X** |  |
| Secondary diagnoses | **X** |  |  | **X** |  |  |  |  |  |  | **X** |  | **X** |  |
| Comorbidities | **X** |  |  |  |  |  |  |  |  |  |  | **X** | **X** |  |
| Charlson co-morbidity index score | **X** |  |  |  |  |  |  |  |  |  |  | **X** | **X** |  |
| Observations |  |  |  |  |  |  |  |  |  |  |  |  |  |  |
| Heart rate (bpm) | **X** |  |  |  |  |  |  |  |  |  | **X** |  | **X** |  |
| Blood pressure (mmHg) | **X** |  |  |  |  |  |  |  |  |  | **X** |  | **X** |  |
| Respiratory rate (breaths/min) | **X** |  |  |  |  |  |  |  |  |  | **X** |  | **X** |  |
| Supplemental oxygen (Yes/no) | **X** |  |  |  |  |  |  |  |  |  | **X** |  | **X** |  |
| **Supplementary Material 2: Study procedures, timepoints and data sources for the UNIVERSAL Study** | | | | | | | | | | | | | | |
| **Study visit** | **Visit 1** | **Visit 2** | **Visit 3** | **Visit 4** |  |  | **Visit 5** |  | **Visit 6** | **Visit 7** |  | **Data source** | | |
| **Time (days):** | **Day 1** | **Day 3** | **Day 7** | **Discharge** | **Week 2** | **Week 4** | **Week 6** | **Week 8** | **Week 12** | **Week 26** | **Readmission** | **Participant** | **Medical records** | **BioFire® FilmArray® System** |
| **Window** |  | **+/- 1 day** | **+/- 1 day** | **+/- 3 days** |  |  |  |  |  |  |  |  |  |  |
| Oxygen delivery device | **X** |  |  |  |  |  |  |  |  |  | **X** |  | **X** |  |
| Fraction of inspired oxygen (% or L/min) | **X** |  |  |  |  |  |  |  |  |  | **X** |  | **X** |  |
| Oxygen saturation (%) | **X** |  |  |  |  |  |  |  |  |  | **X** |  | **X** |  |
| Temperature (^o^C) | **X** |  |  |  |  |  |  |  |  |  | **X** |  | **X** |  |
| Investigations |  |  |  |  |  |  |  |  |  |  |  |  |  |  |
| Blood results | **X** |  |  | **X** |  |  |  |  |  |  |  |  | **X** |  |
| Viral PCR testing result (from standard of care) | **X** |  |  |  |  |  |  |  |  |  |  |  | **X** |  |
| BioFire® FilmArray® respiratory panel result | **X** |  |  |  |  |  |  |  |  |  |  |  |  | **X** |
| RALE score (calculated by clinician) | **X** |  |  |  |  |  |  |  |  |  |  |  |  |  |
| Additional microbiology results |  |  |  | **X** |  |  |  |  |  |  |  |  | **X** |  |
| Admission chest x-ray report |  |  |  | **X** |  |  |  |  |  |  |  |  | **X** |  |
| CT chest report (if done) |  |  |  | **X** |  |  |  |  |  |  |  |  | **X** |  |
| Discharge details |  |  |  |  |  |  |  |  |  |  |  |  |  |  |
| Discharge diagnosis |  |  |  | **X** |  |  |  |  |  |  |  |  | **X** |  |
| Date of discharge (length of stay in days) |  |  |  | **X** |  |  |  |  |  |  |  |  | **X** |  |
| HRG codes |  |  |  | **X** |  |  |  |  |  |  |  |  | **X** |  |
| Measures of safety/severity/mortality/recovery |  |  |  |  |  |  |  |  |  |  |  |  |  |  |
| Ordinal scale for clinical improvement (OSCI)^1^ | **X** | **X** | **X** | **X** |  |  | **X** |  | **X** | **X** | **X** |  | **X** |  |
| ICU/HDU admission and duration |  |  |  | **X** |  |  |  |  |  |  |  |  | **X** |  |
| Number of organs supported |  |  |  | **X** |  |  |  |  |  |  |  |  | **X** |  |
| Death within 30 days of enrolment |  |  |  | **X** |  |  | **X** |  |  |  |  |  | **X** |  |
| Death within 60 days of enrolment |  |  |  | **X** |  |  |  |  | **X** |  |  |  | **X** |  |
| Readmission within 30 days |  |  |  | **X** |  |  | **X** |  |  |  |  |  | **X** |  |
| Treatments/assessments during admission |  |  |  |  |  |  |  |  |  |  |  |  |  |  |
| Invasive ventilation during admission |  |  |  | **X** |  |  |  |  |  |  |  |  | **X** |  |
| CPAP/NIV therapy |  |  |  | **X** |  |  |  |  |  |  |  |  | **X** |  |
| Long term oxygen therapy assessment |  |  |  | **X** |  |  |  |  |  |  |  |  | **X** |  |
| Nicotine replacement therapy |  |  |  | **X** |  |  |  |  |  |  |  |  | **X** |  |
| **Supplementary Material 2: Study procedures, timepoints and data sources for the UNIVERSAL Study** | | | | | | | | | | | | | | |
| **Study visit** | **Visit 1** | **Visit 2** | **Visit 3** | **Visit 4** |  |  | **Visit 5** |  | **Visit 6** | **Visit 7** |  | **Data source** | | |
| **Time (days):** | **Day 1** | **Day 3** | **Day 7** | **Discharge** | **Week 2** | **Week 4** | **Week 6** | **Week 8** | **Week 12** | **Week 26** | **Readmission** | **Participant** | **Medical records** | **BioFire® FilmArray® System** |
| **Window** |  | **+/- 1 day** | **+/- 1 day** | **+/- 3 days** |  |  |  |  |  |  |  |  |  |  |
| Respiratory education during admission |  |  |  | **X** |  |  |  |  |  |  |  |  | **X** |  |
| Patient-reported outcomes (PROs) |  |  |  |  |  |  |  |  |  |  |  |  |  |  |
| FLU-PRO PLUS | **X** | **X** | **X** |  | **X** | **X** |  | **X** | **X** |  |  | **X** |  |  |
| EQ-5D-5L | **X** | **X** | **X** | **X^2^** | **X** | **X** |  | **X** | **X** | **X** |  | **X** |  |  |
| GAD-7 |  |  |  | **X** |  |  | **X** |  | **X** | **X** |  | **X** |  |  |
| PHQ-9 |  |  |  | **X** |  |  | **X** |  | **X** | **X** |  | **X** |  |  |
| FACIT fatigue score |  |  |  | **X** |  |  | **X** |  | **X** | **X** |  | **X** |  |  |
| Clinical samples taken |  |  |  |  |  |  |  |  |  |  |  |  |  |  |
| Blood for plasma analysis (EDTA 4mL) | **X** | **X** |  |  |  |  |  |  |  |  |  |  |  |  |
| Blood for serum analysis (4mL) | **X** | **X** | **X** |  |  |  |  |  |  |  |  |  |  |  |
| Blood for DNA analysis (8.5 mL PAXgene) | **X** | **X** |  |  |  |  |  |  |  |  |  |  |  |  |
| Blood for RNA analysis (2.5 mL PAXgene) | **X** | **X** |  |  |  |  |  |  |  |  |  |  |  |  |
| Nose and throat swab in viral transport media | **X** | **X** |  |  |  |  |  |  |  |  |  |  |  |  |
| Nasal swab in Amies media | **X** |  |  |  |  |  |  |  |  |  |  |  |  |  |
| Naso-sorption wick | **X** |  |  |  |  |  |  |  |  |  |  |  |  |  |
| Follow up/recovery data |  |  |  |  |  |  |  |  |  |  |  |  |  |  |
| New investigations |  |  |  |  |  |  | **X** |  | **X** | **X** |  | **X** | **X** |  |
| Healthcare Utilisation |  |  |  |  |  |  | **X** |  | **X** | **X** |  | **X** | **X** |  |
| Absence from work/college and duration |  |  |  |  |  |  | **X** |  | **X** | **X** |  | **X** |  |  |
| Medication and changes to medication |  |  |  |  |  |  |  |  |  |  |  |  |  |  |
| Antibiotics | **X** |  |  | **X** |  |  | **X** |  | **X** | **X** |  |  | **X** |  |
| Anti-viral therapy | **X** |  |  | **X** |  |  | **X** |  | **X** | **X** |  |  | **X** |  |
| Systemic steroids | **X** |  |  | **X** |  |  | **X** |  | **X** | **X** |  |  | **X** |  |
| Inhaled therapy | **X** |  |  | **X** |  |  | **X** |  | **X** | **X** |  |  | **X** |  |
| Nebulised therapy | **X** |  |  | **X** |  |  | **X** |  | **X** | **X** |  |  | **X** |  |
| Oxygen | **X** |  |  | **X** |  |  | **X** |  | **X** | **X** |  |  | **X** |  |
| Immunosuppressive therapy | **X** |  |  | **X** |  |  | **X** |  | **X** | **X** |  |  | **X** |  |
| Biological therapy | **X** |  |  | **X** |  |  | **X** |  | **X** | **X** |  |  | **X** |  |

**Supplementary Material 2**: Study procedures, timepoints and data sources for the UNIVERSAL Study. Data is collected at multiple timepoints from admission through to six months post-discharge, capturing detailed demographic, clinical, and investigational information. ^1^OSCI score collected daily during admission. ^2^Required if not already completed when discharge is on day 3 or 7 of enrolment. Data collection variables, timepoints of collection and data sources for UNIVERSAL study. BMI, Body mass index; CPAP, continuous positive airway pressure; EQ-5D-5L, EuroQol 5-Dimension 5-Level; FACIT, functional assessment of chronic illness – fatigue scale; FLU-PRO PLUS, inFLUenza Patient-Reported Outcome Plus; GAD-7, Generalised anxiety disorder-7; HDU, High dependency unit; HRG, Healthcare resource group; ICU, Intensive care unit; PHQ-9, Patient health questionnaire-9; RALE, Radiographic assessment of lung edema.
