# Supplementary material for: Understanding Infection, Viral Exacerbation and Respiratory Symptoms at Admission-Longitudinal (UNIVERSAL) study: a prospective observational cohort study protocol
Source: BMJ Open. 2025 Apr 9;15(4):e093427. doi: 10.1136/bmjopen-2024-093427 (PMC11987089; doi:10.1136/bmjopen-2024-093427)
Supplement: Supplementary file 3 [file bmjopen-2024-093427.reviewer_comments.pdf]

PEER REVIEW HISTORY

BMJ Open publishes all reviews undertaken for accepted manuscripts. Reviewers are asked to complete a checklist review form and are provided with free text boxes to elaborate on their assessment. These free text comments are reproduced below.

ARTICLE DETAILS

Title (Provisional)

Understanding Infection, Viral Exacerbation and Respiratory Symptoms at Admission-Longitudinal (UNIVERSAL) Study: A Prospective Observational Cohort Study Protocol

Authors

Morelli, Tommaso; Purcell, Martha; Rodrigues, Pedro; Roberts, Charles; Cox, Olivia; Lee, Paul H; Thorne, Kerensa; Allen, Alexander; Cazaly, Angelica; Nuttall, Jacqui; Raftery, James; Griffiths, Gareth; Cook, Andrew; White, Nicola; Greening, Neil J; Pavitt, Matthew; Myerson, James; Marciniak, Stefan J; Daneshvar, Cyrus; Crooks, Michael G; Mitchelmore, Philip; Chalmers, J; Siddiqui, Salman; Staples, Karl J.; clark, tristan William; Freeman, Anna; Wilkinson, Tom

---

VERSION 1 - REVIEW

**Reviewer**	**1**
**Name**	**Wi, Chung-Il**
**Affiliation**	**Mayo Clinic, Pediatric and Adolescent Medicine**
**Date**	**02-Oct-2024**
**COI**	**None**

This protocol of a prospective longitudinal observational study was well-written and very timely to answer clinically relevant questions post-Covid era. I recommend accept after clarification and minor revision.

1. One of the primary endpoints is to study the impact of different RVIs. It's not clear impact on what. On page 12, it reads documenting the occurrence of various rep. viruses. Is this referring simply to "which" virus was infected?

2. Inclusion criteria: While Positive test result is one of inclusion criteria, the protocol says "a subset of respiratory virus negative" participants will be used as a control group. This is confusing. Reviewer thought that only positive-tested patients will be approached to include in this study. Please clarify.

3. It's not clear how test-negative patients will be used as controls. Test-negative patients who have respiratory symptoms for admission are likely to be other respiratory virus test

positive, but not detected by BioFire FilmArray. Please include which viruses will be detected by BioFire FilmArray.

4. Socioeconomic status was once mentioned to be collected. Please clarify which standardized measure of SES will be used. While SES is very strong predictors for respiratory infection outcomes, it is not included in multivariate analysis in the analytic plan. Please justify.

5. Reviewer suggests reporting comparison of available data between study participants vs. non-participants who do not consent.

VERSION 1 - AUTHOR RESPONSE

Reviewer: 1
Dr. Chung-Il Wi, Mayo Clinic
Comments to the Author:
This protocol of a prospective longitudinal observational study was well-written and very timely to answer clinically relevant questions post-Covid era. I recommend accept after clarification and minor revision.

1. One of the primary endpoints is to study the impact of different RVIs. It's not clear impact on what. On page 12, it reads documenting the occurrence of various rep. viruses. Is this referring simply to "which" virus was infected?

Thank you for your comment. To clarify, the primary objective of the study is not only to document which respiratory viruses are present but also to characterise their clinical impact on hospitalised adults. We have refined the text to explicitly state that the study assesses the association of various respiratory viral infections (RVIs) with disease severity, progression, and recovery, rather than simply identifying their presence. Additionally, we have clarified that the study is not limited to SARS-CoV-2, influenza, and RSV but also includes a broader range of respiratory viruses, such as rhinoviruses, parainfluenza viruses, and human metapneumovirus.

The revised text For Primary outcomes on page 12 now reads:

The primary outcomes of the UNIVERSAL study are focused on characterising the clinical impact of respiratory viral infections (RVIs) in hospitalised adults. This includes not only documenting the occurrence of various respiratory viruses, including influenza, respiratory syncytial virus (RSV), and SARS-CoV-2, but also a broader range of viruses such as rhinoviruses, parainfluenza viruses, and human metapneumovirus. The study aims to evaluate their association with disease severity, progression, and recovery. The study also aims to identify clinical and biological predictors of these outcomes including markers such as viral load, inflammatory markers such as C-reactive protein (CRP), and patient

demographics. Another key outcome is measuring the duration of hospital stay for patients with RVIs and understanding the factors that contribute to prolonged hospitalisation.

2. Inclusion criteria: While Positive test result is one of inclusion criteria, the protocol says "a subset of respiratory virus negative" participants will be used as a control group. This is confusing. Reviewer thought that only positive-tested patients will be approached to include in this study. Please clarify.

Thank you for your comment. To clarify, the primary study population consists of patients who test positive for a respiratory virus on the BioFire FilmArray PCR panel. However, as an additional component of the study, a small subset (n=100) of patients who test negative on the BioFire panel will be recruited for sampling and follow-up. The purpose of this group is to serve as a comparison cohort, allowing us to explore differences in clinical characteristics and outcomes between patients with confirmed respiratory viral infections and those in whom no virus was detected.

All participants, including those in the virus-negative group, provide written informed consent before enrolment (see UNIVERSAL ICF – Supplementary Material 1). Additionally, this amendment to include virus-negative participants was approved in Amendment 5 of the study protocol (Version 6, 20th September 2023).

To avoid confusion, we have adjusted the wording in the protocol to clarify that the primary study population is virus-positive patients, while the test-negative group is a small exploratory arm.

The following short section has been added on page 9

Virus-negative comparison cohort:

While the primary focus of the UNIVERSAL study is on hospitalised patients who test positive for a respiratory virus using the BioFire® FilmArray® System, a subset of virus-negative patients (n=100) is also being recruited, (as per protocol amendment version 6.0, 20th September 2023). This group consists of individuals who present with acute respiratory illness but test negative for all viruses. This comparison cohort will allow for exploratory analyses of differences in clinical characteristics and outcomes between patients with confirmed viral infections and those in whom no virus is detected. All virus-negative participants provide written informed consent (see Supplementary Material 1).

3. It's not clear how test-negative patients will be used as controls. Test-negative patients who have respiratory symptoms for admission are likely to be other respiratory virus test positive, but not detected by BioFire FilmArray. Please include which viruses will be detected by BioFire FilmArray.

The test-negative subset was included as an exploratory comparison group as described above. We acknowledge that some patients who test negative on the BioFire panel may have respiratory infections caused by pathogens not detected by this assay, or non-viral causes of

illness. This subset was recruited to provide a reference point for evaluating clinical features and outcomes in patients without a confirmed viral diagnosis.

For clarity, the BioFire FilmArray system detects the following respiratory viruses: Influenza A, Influenza B, RSV, SARS-CoV-2, Parainfluenza viruses 1-4, Human metapneumovirus, Rhinovirus/enterovirus, Adenovirus and Coronaviruses 229E, NL63, OC43, HKU1.

We have now explicitly listed these viruses in the protocol on page 9 to clarify the scope of viral testing.

4. Socioeconomic status was once mentioned to be collected. Please clarify which standardized measure of SES will be used. While SES is very strong predictors for respiratory infection outcomes, it is not included in multivariate analysis in the analytic plan. Please justify.

Thank you for your comment. We acknowledge the importance of socioeconomic status (SES) in relation to respiratory infection outcomes. However, in this study, SES is not a primary variable of interest, and we are not employing a formal standardized measure of SES. Instead, we are collecting participant postcodes, which will allow linkage to area-level deprivation indices (such as the Index of Multiple Deprivation (IMD)) in future analyses.

To address your concern, we have now clarified in the protocol that SES will be included as a covariate in the multivariate analysis, alongside other key covariates such as vaccination status and comorbidities. We will assess multicollinearity among covariates using appropriate statistical methods (e.g., Variance Inflation Factor (VIF)) to ensure that highly correlated variables do not distort model estimates.

To improve clarity, we have updated the manuscript to specify the nature of SES data collection and its role in the analysis plan. Additionally, we have revised the protocol to ensure alignment with the study's primary objectives and to prevent any ambiguity regarding the interpretation of SES in the analysis.

The following short section has been added on page 19 to clarify SES data collection:

While socioeconomic factors are not a primary focus of the study, participant postcodes are collected to allow for area-based deprivation analyses (e.g., linkage to the Index of Multiple Deprivation (IMD)).

The statistical methods section on page 23 has been updated to reflect the inclusion of SES in multivariable analyses and now reads:

Multivariable regression models will be employed to identify associations between key predictors and outcomes while controlling for potential confounders. These models will be adjusted for demographic factors (age, gender, ethnicity, socioeconomic status), clinical variables (severity scores, comorbidities), and treatment regimens. Prior to model fitting, multicollinearity among covariates will be assessed using appropriate statistical methods

(e.g., Variance Inflation Factor (VIF)), and adjustments will be made if necessary to ensure model stability.

5. Reviewer suggests reporting comparison of available data between study participants vs. non-participants who do not consent.

Thank you for this suggestion. However, we do not collect or retain data on individuals who decline participation in the UNIVERSAL study, and as such, a formal comparison between participants and non-participants is not possible. We acknowledge that this may introduce potential selection bias, and we will discuss this as a limitation in the study's interpretation.
